# Fostering engagement in the digital age: the mediating role of self-efficacy and self-regulation between enjoyment and learner engagement in AI-assisted EFL writing

**DOI:** 10.3389/fpsyg.2026.1848701

**Published:** 2026-06-15

**Authors:** Xiaojing Li, Tao Wang, Wei Wang, Lei Liu

**Affiliations:** 1 School of International Studies, Zhengzhou University, Zhengzhou, China; 2 School of International Exchange, Shandong Vocational University of Foreign Affairs, Weihai, China

**Keywords:** AI-assisted writing, enjoyment, learner engagement, self-efficacy, self-regulated learning strategies

## Abstract

**Introduction:**

The development of artificial intelligence (AI) has created new opportunities for AI-supported foreign language teaching and applications. This study investigates the interrelationships among foreign language enjoyment (FLE), writing self-efficacy, self-regulated learning (SRL) strategies, and learner engagement within the context of AI-assisted English as a foreign language (EFL) writing.

**Methods:**

A cross-sectional survey design was employed, involving 535 Chinese university students with prior experience in AI-assisted writing. Participants completed adapted and validated scales measuring FLE, writing self-efficacy, SRL strategies, and learner engagement. Structural equation modeling (SEM) was used to test a hypothesized partial mediation model linking these constructs.

**Results:**

The results supported a well-fitting partial mediation model. FLE was found to be a direct and significant positive factor of learner engagement. Furthermore, the analysis confirmed that self-efficacy and SRL strategies act as sequential mediators in the relationship between FLE and engagement. Specifically, FLE is positively associated with learners' self-efficacy, which in turn promotes the use of SRL strategies, ultimately leading to deeper cognitive, behavioral, emotional, and agentic engagement in the writing task.

**Discussion:**

The study extends Control-Value Theory, Broaden-and-Build Theory, Social Cognitive Theory, and Self-Regulated Learning Theory to the novel domain of AI-assisted language learning, highlighting the critical affective and cognitive pathways that foster engaged learning. Pedagogical implications are discussed, emphasizing the importance of designing enjoyable, confidence-building, and strategy-rich AI-assisted writing environments.

## 1 Introduction

With the rapid development of artificial intelligence (AI), its application has become increasingly prevalent across various sectors, particularly in the field of education and policy (Dwivedi et al., 2021). AI has been conceptualized in multiple ways across different research traditions. For instance, Russell and Norvig (2016) define AI as systems that emulate human cognitive functions such as learning and problem-solving, whereas Kaplanand Haenlein (2019) view AI as systems capable of adapting flexibly to achieve user objectives. Rather than representing a single unified definition, these two perspectives offer complementary emphases—one focusing on cognitive simulation and the other on adaptive goal-oriented behavior—both of which are relevant to understanding how AI functions as a collaborative tool in educational contexts. Accordingly, AI is markedly reshaping the way English as a foreign language (EFL) is learned, especially in writing (Lee Y. J. et al., 2025). The AI-assisted writing is a collaborative process where a human writer uses an AI tool as a partner to set goals, generate, organize, or refine texts, while maintaining final creative control (Gero and Chilton, 2019; Jakesch et al., 2023; Nguyen et al., 2024; Salvagno et al., 2023). To enhance the effectiveness of English learning and achieve efficient completion of academic writing tasks in English, undergraduate, postgraduate, and doctoral students in Chinese universities benefit from AI-assisted writing tools through features such as language suggestions and structural scaffolding, thus constituting the main group using AI to support English learning and writing. This collaborative partnership between learners and AI can enhance writing outcomes across multiple dimensions. From a cognitive perspective, AI tools have been shown to improve textual accuracy and coherence by providing linguistic feedback and scaffold writing instruction (Al Fraidan, 2025; Li and Wilson, 2025; Moltudal et al., 2025). From an affective perspective, AI-assisted writing environments can foster learners' foreign language confidence (Zhang et al., 2025). From a motivational and behavioral perspective, such collaboration has been found to boost writing self-efficacy, strategy use, and behavioral intention (Almusharraf et al., 2025).

Recent studies tend to examine four variables within technology-enhanced language learning environments, providing valuable insights into the relationships among enjoyment, self-efficacy, self-regulated learning (SRL) strategies, and learner engagement. Firstly, Wei (2023) demonstrates that AI-mediated instruction can affect learners' self-regulated learning. Some researchers find that enjoyment is a significant antecedent of writing self-efficacy (Zhang L. J. et al., 2025) and of SRL strategies (Dan and Bai, 2025). Some validate self-efficacy as a factor of both SRL strategies (Teng, 2024) and engagement (Pan, 2022; Tsao, 2021). Of all four variables, engagement is widely recognized as a key contributing factor in successful second language learning (Pearson, 2024), as it is closely associated with the quality of English writing outcomes. During the writing process, L2 learners may experience varying degrees of foreign language enjoyment (FLE), which can further influence their engagement.

Derakhshan and Fathi (2024) find that enjoyment in online settings is positively linked to self-efficacy and learner engagement, supporting the FLE-self-efficacy link and the FLE-engagement link. Further, (Wang and Wang 2025a) show that enjoyment is positively related to self-efficacy and learner engagement, with self-efficacy serving as a mediator. Xu et al. (2024) report that self-efficacy fully mediates the relationship between SRL strategies and engagement in smart classrooms, supporting the sequential pathway SRL strategies → self-efficacy → engagement. These findings suggest FLE influences engagement through self-efficacy and through SRL strategies.

Based on the above research background, this study finds that enjoyment, self-efficacy, and SRL strategies are interconnected and collectively affect learner engagement, with self-efficacy and SRL strategies serving as mediators. The purpose of this study is to test the hypothesized structural model in a new context of AI-assisted EFL writing. This provides clear evidence for the direct and indirect pathways in AI-assisted EFL writing.

The research questions of this study are as follows:

(1) To what extent are enjoyment, self-efficacy, and SRL strategies associated with learner engagement in AI-assisted EFL writing?

(2) Do self-efficacy and SRL strategies sequentially mediate the relationship between enjoyment and learner engagement in AI-assisted EFL writing?

## 2 Literature review and hypotheses

Research has confirmed the associated pathway from FLE to learner engagement (Derakhshan and Yin, 2025; Hosseini et al., 2022). However, there is still a theoretical and empirical limitation which persists. Prior structural equation modeling (SEM) research neglects to integrate those established pathways into a comprehensive structural model that positions FLE as a distal factor and learner engagement as a final outcome through the cognitive and motivational mechanisms of self-efficacy and SRL strategies. Therefore, the hypothesized structural model remains theoretically and empirically unverified within a unified framework.

### 2.1 Theoretical framework

Control-Value Theory (CVT) explains how emotions arise in academic settings. According to Pekrun (2006), two key factors matter, including learners' perceived control over activities and the subjective value they assign to those activities (Pekrun, 2006, pp. 315–317). Based on CVT, Pekrun concluded that positive emotions, such as enjoyment, are positively associated with individuals' intrinsic and extrinsic motivation, and facilitate the self-regulation and the use of creative learning strategies. Enjoyment is essentially a positive emotion arising from high control and high value. In turn, enjoyment is positively associated with self-efficacy and prompts individuals to proactively manage their learning process by employing a variety of self-regulated learning strategies. At the same time, Social Cognitive Theory (SCT) holds the viewpoint that self-efficacy is a vital antecedent to self-regulation, as learners with robust confidence are more resilient and strategic in learning (Bandura, 1997). Broaden-and-Build Theory (BBT) holds the viewpoint that positive emotions can broaden individuals' cognitive and behavioral repertoires, encouraging engagement in activities (Fredrickson, 2004). Among the positive emotions, such as love, pride, and enjoyment, experienced by EFL learners, enjoyment has received the most attention from second language learning researchers (Dewaele and MacIntyre, 2019) and has been demonstrated to be a strong factor in the EFL process (Pekrun et al., 2007; Zhang and Tsung, 2021).

According to the core tenets of SCT, there is a reciprocal interaction among the individual, behavior, and the environment. Individuals' beliefs, such as self-efficacy, promote the adoption of self-regulated learning behaviors, which in turn enable them to actively shape their learning environment. More specifically, Self-Regulated Learning Theory (SLT) (Zimmerman, 1986, 2000) posits that learning is a cyclical process in which individuals proactively regulate their own learning. Learners with high self-efficacy are more likely to complete a full self-regulatory cycle: They actively regulate their learning strategies and engage in self-reflection after learning, thereby completing the learning cycle and further optimizing their learning strategies.

Through the interaction of these factors, learners are thus enabled to complete their learning activities.

### 2.2 Enjoyment

Based on CVT, the research conducted by Derakhshan and Yin (2025) inspects the association between positive emotions and academic engagement among Chinese and Iranian EFL students, showing that pride, hope, and enjoyment can be positively related to the students' academic engagement. Dai and Wang (2024) investigate the intricate relationships between Chinese EFL learners' enjoyment and their engagement within technology-enhanced environments. Their findings reveal that enjoyment is significantly associated with technology integration and academic engagement. Hosseini et al. (2022) further support this finding. Their structural model shows that FLE is a strong factor of engagement. Moreover, the study reveals that both ideal L2 writing self and writing enjoyment significantly impact L2 writing self-efficacy directly (Zhang L. J. et al., 2025). Based on the above findings, this study proposes the following hypothesis in the context of AI-assisted EFL writing:

H1: Enjoyment is positively associated with engagement within AI-assisted EFL writing;

Zhao et al. (2023) demonstrate that writing enjoyment is positively related to greater use of writing strategies. Dan and Bai (2025) find that positive teacher-student and peer relationships promote enjoyment, which increases SRL strategies use. Furthermore, Luo and Wang (2023) confirm that FLE positively correlates with SRL strategies, whereas foreign language boredom negatively relates to it, with SRL strategies mediating the effects of both emotions on learning effectiveness.

Given these findings, we propose the following hypotheses in the context of AI-assisted EFL writing:

H2: Enjoyment is positively associated with self-efficacy within AI-assisted EFL writing;

H3: Enjoyment is positively associated with SRL strategies within AI-assisted EFL writing;

### 2.3 Self-efficacy and SRL strategies

Self-efficacy refers to “beliefs in one's capabilities to organize and execute the courses of action required to produce given attainments” (Bandura, 1997, p. 3). These beliefs are shaped by four key sources: enactive mastery experiences; vicarious experiences gained through social comparison and observation; verbal persuasion and other social influences that affirm competence; and physiological and affective states that inform self-assessment. Students' self-efficacy beliefs—particularly their belief in their ability to regulate learning and master academic tasks—strongly influence their aspirations, motivation, and eventual academic achievements (Bandura, 1993).

Writing self-efficacy is defined as a learner's beliefs about their own writing capabilities (Schunk and Swartz, 1993). Research in blended environments demonstrates that self-efficacy is not only directly positively associated with SRL but is also indirectly positively associated with it through positive learning behaviors (Chen et al., 2024). In specific L2 writing contexts, self-efficacy is a strong factor in the use of cognitive, metacognitive, and motivational SRL strategies (Teng, 2024). Research in AI-assisted writing confirms that both writing self-efficacy and task-specific AI self-efficacy are significantly and positively associated with the adoption of SRL strategies (Jin et al., 2025). Furthermore, in generative AI-supported contexts, self-efficacy is closely linked to strategic learning behaviors (Liu and Zhang, 2025).

Building on the evidence above, the following hypothesis is advanced:

H4: Self-efficacy is positively associated with SRL strategies within AI-assisted EFL writing;

What's more, Derakhshan and Fathi (2024) find that online learning self-efficacy is both a direct factor and a mediator between positive traits and online engagement. Xu et al. (2024) identify that SRL strategies influence multifaceted engagement only through the pathway of self-efficacy. This connection extends to learner wellbeing, as Cong et al. (2024) report that academic self-efficacy boosts engagement. Zare et al. (2025) find that L2 writing self-efficacy is positively related to learner engagement through SEM. These studies highlight that self-efficacy is positively associated with engagement. Research has demonstrated that enjoyment is positively related to self-efficacy through fostering positive emotions, intrinsic motivation, and perceived competence (Schukajlow et al., 2012).

Based on the above, this study proposes the following hypothesis in the context of AI-assisted EFL writing:

H5: Self-efficacy is positively associated with engagement within AI-assisted EFL writing;

SRL refers to the proactive and cyclical process in which learners autonomously set goals for their learning, and then actively monitor and adjust their cognition, motivation, and behavior in order to achieve goals (Zimmerman, 1986). Building on this concept, SRL strategy is defined as “actions and processes directed at acquisition of information or skills that involve agency, purpose, and instrumentality perceptions by learners” (Zimmerman, 2010, p. 5). In the context of writing, 16 kinds of SRL strategies in writing have been identified by Graham and Harris (2000), like goal setting and planning, seeking information, self-monitoring, and self-evaluating. Cleary et al. (2021) identify that students possessing strong SRL strategies tend to exhibit higher engagement. Zare et al. (2024) find that SRL strategies use was a strong positive factor of task engagement among university EFL learners, with qualitative data underscoring the role of multifaceted (meta)strategies in sustaining engagement. Shi et al. (2025) further reveal that SRL and task engagement are strongly positively correlated among secondary EFL students in AI-assisted classrooms. Wang X. et al. (2025) examine the impact of an automated writing evaluation (AWE) tool on engagement in persuasive writing among Chinese EFL learners, indicating that AWE tool can promote deeper metacognitive engagement in the writing process.

**Figure 1:**
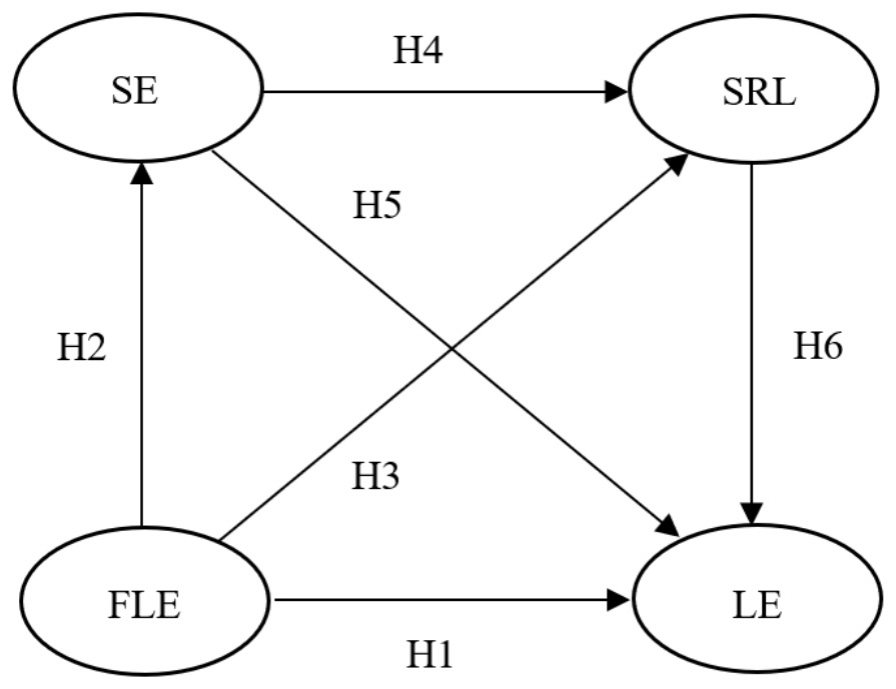
Hypothesized model.

Based on the above, this study proposes the following hypothesis in the context of AI-assisted EFL writing, and the overall theoretical model is shown in Figure 1:

H6: SRL strategies are positively associated with engagement within AI-assisted EFL writing.

CVT provides the affective foundation, BBT demonstrates the emotion-engagement link, and SCT and SLT supply the cognitive-regulatory mechanism. On the basis of integrating CVT, BBT, SCT, and SLT, the present study aims to investigate the pathways among the four variables illustrated in Figure 1 within the new context of AI-assisted writing—that is, the six hypotheses proposed above—so as to explore whether, in the new context of AI, the relationships among these four variables may yield pathways that differ from the hypotheses derived from the existing literature.

## 3 Methodology

### 3.1 Participants

This study employed a convenience sampling strategy to recruit participants from universities in China. Recruitment announcements were disseminated via popular social media platforms (WeChat, QQ, and RedNote) between January and March 2026. To ensure the relevance of the data to the research focus on AI-assisted writing, specific inclusion criteria were established: (1) current enrollment in a university-level English course; (2) completion of at least six years of formal English education; and (3) demonstrable prior experience in using AI tools (e.g., ChatGPT, Doubao) for English writing tasks.

### 3.2 Instruments

The instrument section consists of five parts: Demographic information and four separate scales. To ensure measurement consistency and enhance data accuracy, this study unified all instruments under a single 7-point Likert scale, ranging from “1 (strongly disagree)” to “7 (strongly agree)”. While the original scales varied in their response formats—specifically, the Foreign Language Enjoyment Scale (Li et al., 2018) used a 5-point scale, the Writing Self-Regulated Learning Strategies Scale (Sun and Wang, 2020) used a 4-point scale, and both the Writing Self-Efficacy Scale (Sun and Wang, 2020) and the Learner Engagement Scale (Reeve and Tseng, 2011) originally employed 7-point scales—all items were standardized to the 7-point format. This specific scaling decision was informed by prior research indicating that 7-point scales yield stronger correlations with statistical tests (Lewis, 1993), produce more precise data compared to other formats (Srivastava and Rogers, 2021), and offer an optimal balance between reliability, validity, and respondent preference, with diminishing psychometric returns beyond seven points (Colman et al., 1997; Preston and Colman, 2000; Brown, 2000).

#### 3.2.1 Foreign language enjoyment scale

The enjoyment scale was originally from Li et al. (2018) containing 11 items with 3 dimensions (FLE-Private, FLE-Teacher, and FLE-Atmosphere). Necessary adaptations were made. For instance, the original item “I don't get bored” was revised to “I don't get bored with the AI-assisted English writing class.”

#### 3.2.2 Writing self-efficacy scale

The scale of English writing self-efficacy was originally from Sun and Wang (2020), consisting of 5 categories: ideation (3 items), organization (5 items), grammar and spelling (4 items), use of English writing (8 items), and self-efficacy for self-regulation (7 items). Necessary adaptations were made. For instance, the original item “I can think of many ideas for my writing” was revised to “I can use artificial intelligence to think of many ideas for my English writing.”

#### 3.2.3 Writing self-regulated learning strategies scale

The scale of writing self-regulated learning strategies was originally from Sun and Wang (2020), including 3 categories: environmental SRL strategies (8 items); behavioral SRL strategies (8 items); and personal SRL strategies (10 items). Necessary adaptations were made. For example, the original item “Write an outline before writing English compositions” was revised to “Write an outline before writing AI-assisted English compositions.”

#### 3.2.4 Learner engagement scale

The learning engagement scale originally came from Reeve and Tseng (2011), containing 22 items with 4 dimensions (agentic engagement, behavioral engagement, emotional engagement, and cognitive engagement). Necessary adaptations were made. For instance, the original item “During class, I ask questions” was revised to “During AI-assisted English writing class, I ask questions.”

It should be noted that the modifications made to the original scales were strictly limited to wording changes to fit the AI-assisted writing context. Crucially, the original dimensional structure of all four scales was strictly preserved. No items were deleted or re-categorized. To ensure content validity, the adapted scales were reviewed by 4 experts in applied linguistics and L2 writing (2 professors and 2 associate professors). They confirmed that the adapted items accurately reflected the target constructs within the AI writing context and that the dimensional structure remained intact.

The English version of the scales was translated into Chinese to help participants understand it correctly and clearly. To ensure content accuracy, the translated scales were translated back into English and compared with the original version, with the help of two experts in applied linguistics.

### 3.3 Procedure

The study was approved by the Institutional Review Board of the authors' institution. Prior to completing the questionnaire, the respondents were informed of the purpose and use of the research, and were assured that the data and personal information would only be used for this study. The respondents took part in the experiment voluntarily and anonymously. A small monetary compensation from personal living funds was provided to the participants. There is no external or financial support from any research fund involved in this compensation.

### 3.4 Data collection

Data were collected between January 2026 and March 2026 using the online platform “wjx.cn”. The questionnaire link was distributed via WeChat, QQ, and RedNote. No personally identifiable information was collected. Before completing the questionnaire, respondents were informed of the study's purpose and use.

Of the 719 collected responses, 184 were excluded from the analysis. Among these, 63 were removed because the respondents reported no prior experience using AI to assist their English writing, which did not meet the experimental inclusion criteria. The remaining 121 were excluded due to poor response quality, characterized by notably short completion times (responses with a total completion time falling below two standard deviations below the mean (Mean – 2 × SD)), highly uniform answer patterns (the exact same response option for every item across all four measurement scales), or incomplete questionnaire responses. Consequently, 535 valid responses were retained, yielding a valid response rate of 74.4%.

### 3.5 Data analysis

SPSS 27.0 and AMOS 24.0 were used for the data analysis. SPSS 27.0 was first employed to compute descriptive statistics, internal consistency (Cronbach's α), and Harman's single-factor test for common method bias. Subsequently, AMOS 24.0 was utilized to conduct confirmatory factor analysis (CFA) to assess convergent and discriminant validity, followed by SEM to evaluate model fit indices. Finally, a bootstrapping procedure with 5,000 resamples was performed in AMOS 24.0 to test the significance of direct, indirect, and total effects, thereby examining the hypothesized pathways.

## 4 Results

### 4.1 Demographic analysis

Table 1 illustrates the demographic features of the sample, which shows a female majority (62.43%, *n* = 334) compared to males (37.57%, *n* = 201). In terms of education background, the vast majority were undergraduates (89.35%, *n* = 478), followed by graduate students (10.09%, *n* = 54) and doctoral students (0.56%, *n* = 3). Regarding academic disciplines, science and engineering accounted for the largest share (40.37%, *n* = 216), followed by humanities and social sciences (35.51%, *n* = 190), economics and management (12.71%, *n* = 68), and medicine (11.40%, *n* = 61).

**Table 1:** Descriptive statistics of participants' demographic information.

Demographics	Classification	Number	Percent (%)
Gender	male	201	37.570
	female	334	62.430
Education background	undergraduate	478	89.346
	graduate	54	10.093
	doctoral students	3	0.561
Academic discipline	science and engineering	216	40.374
	economics and management	68	12.710
	humanities and social sciences	190	35.514
	medicine	61	11.402

Table 2 presents the most frequently used AI tools among respondents. As a multiple-response question, the most frequently used AI tool was Doubao (88.60%, *n* = 474), followed by ChatGPT (29.35%, *n* = 157), Ernie Bot (20.75%, *n* = 111), Tencent Yuanbao (19.44%, *n* = 104), Kimi (13.27%, *n* = 71), Qwen (11.59%, *n* = 62), Gemini (9.53%, *n* = 51), and other tools such as Grammarly, Copy.ai, Quillbot, and others with lower usage rates.

**Table 2:** Frequently used AI tools.

Tools	Number	Percent (%)
Doubao	474	88.598
ChatGPT	157	29.346
Ernie Bot	111	20.748
Tencent Yuanbao	104	19.439
Kimi	71	13.271
Qwen	62	11.589
Gemini	51	9.533
Grammarly	17	3.178
Copy.ai	13	2.430
Quillbot	12	2.243
Others	2	0.374

As shown in Table 3, for weekly time of AI-assisted writing (single-response), over half of the participants reported 1–3 h (54.02%, *n* = 289), followed by 4–6 h (26.73%, *n* = 143), 7–9 h (11.03%, *n* = 59), and more than 9 h (8.22%, *n* = 44).

**Table 3:** Weekly time of AI-assisted writing.

Time	Number	Percent (%)
1–3h	289	54.019
4–6h	143	26.729
7–9h	59	11.028
> 9h	44	8.224

### 4.2 Descriptive statistics

Table A1 in the Appendix presents the results of descriptive statistics analysis conducted on the data of each item. The data of these items have excellent skewness and kurtosis values, according to the criteria suggested by (Kline 2005, p. 50), data can be considered to meet the normality assumption when skewness values range between −3 and +3, and kurtosis values range between −8 and +8. In this study, the absolute values of skewness and kurtosis for all items were less than 1, indicating a well-approximated normal distribution. Therefore, the data are suitable for structural equation modeling analysis.

### 4.3 Common method bias

Consistent with research that relies on self-reported data, the possibility of Common Method Bias (CMB) arises from factors such as consistency motives and social desirability (Podsakoff et al., 2003; Podsakoff and Organ, 1986). Given that CMB can compromise the validity of the findings, this study adopted both procedural and statistical approaches to mitigate and evaluate its influence.

Procedurally, the survey was designed with a focus on respondent anonymity. Several strategies were implemented to reduce CMB, such as counterbalancing item order, randomizing the sequence of questions, and refining scale items to minimize ambiguity. To further alleviate potential bias, different constructs were presented on separate pages, with sufficient rest intervals between pages to reduce respondent fatigue and context carryover effects (Podsakoff et al., 2012).

Statistically, Harman's single-factor test was conducted using principal axis factoring without rotation. The results showed that the first factor accounted for 38.201% of the total variance. This figure falls below the recommended threshold of 40% (Tang and Wen, 2020). Therefore, it can be concluded that common method bias did not significantly affect the findings of this study.

### 4.4 Reliability and validity test

To comprehensively assess construct validity in SEM, composite reliability (CR) and average variance extracted (AVE) were employed as two critical indicators alongside Cronbach's α, McDonald's ω and Guttman's λ2. In SEM, the recommended thresholds are 0.50 for AVE (Fornell and Larcker, 1981), 0.70 for CR and Cronbach's alpha (Hair et al., 2011). As presented in Table A2 and Table 4, all AVE values surpassed 0.701, all CR values exceeded 0.903, all Cronbach's α coefficients exceeded 0.801, all McDonald's ω values exceeded 0.803, and all Guttman's λ2 coefficients exceeded 0.802, indicating that the measurement model demonstrates satisfactory reliability and convergent validity.

**Table 4:** Discriminant validity.

Construct	CR	AVE	MSV	MaxR(H)	SE	LE	SRL	FLE
SE	0.962	0.836	0.666	0.964	**0.914**			
LE	0.903	0.701	0.609	0.919	0.732^***^	**0.838**		
SRL	0.932	0.820	0.666	0.932	0.816^***^	0.775^***^	**0.906**	
FLE	0.914	0.781	0.657	0.947	0.718^***^	0.780^***^	0.811^***^	**0.884**

CR, composite reliability; AVE, average variance extracted; MSV, max shared variance; MaxR(H), maximal reliability; Square roots of AVE are displayed in bold on the diagonal; Off-diagonal values are the Pearson correlations between the constructs. ^***^p < 0.001.

All standardized factor loadings are statistically significant (*p* < 0.001), and most standardized factor loadings were above the recommended threshold of 0.70. Only three standardized factor loadings were below 0.60, with 0.594 (CE8), 0.559 (BS5), and 0.544 (BS3), respectively, which is acceptable according to Hair et al. (2010), who argue that a factor loading of 0.50 is acceptable with large samples. These results demonstrate satisfactory item reliability across the measured constructs.

The assessment of validity encompasses discriminant validity. As illustrated in Table 4, the square root of the AVE for each construct is greater than its corresponding correlations with other constructs, and AVE is greater than its maximum shared variance (MSV) with any other construct, confirming that the measurement model demonstrates adequate discriminant validity (Fornell and Larcker, 1981, p. 46; Grewal et al., 2004, p. 528).

### 4.5 SEM fit indices

The evaluation of model fit was based on multiple fit indices in accordance with established criteria (Bagozzi and Yi, 1988; Hair et al., 2017; Hayduk, 1987; Hu and Bentler, 1998). Following the recommendations of (Kline 2016, p. 269), a comprehensive set of fit statistics is reported, including the chi-square test, degrees of freedom, *p*-value, the Root Mean Square Error of Approximation (RMSEA) along with its 90% confidence interval, the Comparative Fit Index (CFI), and the Standardized Root Mean Square Residual (SRMR).

As presented in Table 5, these indices indicate that the model provides an adequate representation of the observed data and supports the hypothesized relationships among the constructs.

**Table 5:** Model fit indicators.

Fit index	Estimation	Threshold	Source
CMIN	6208.307	–	–
DF	3548	–	–
p	< 0.001	–	–
CMIN/DF	1.750	Between 1 and 3	Hair et al. (2010)
CFI	0.912	>0.900	Bagozzi and Yi (1988)
SRMR	0.046	< 0.080	Hu and Bentler (1998)
RMSEA (90% CI)	0.037 (0.036–0.039)	< 0.080	Hair et al. (2017)

### 4.6 Path analysis

**Figure 2:**
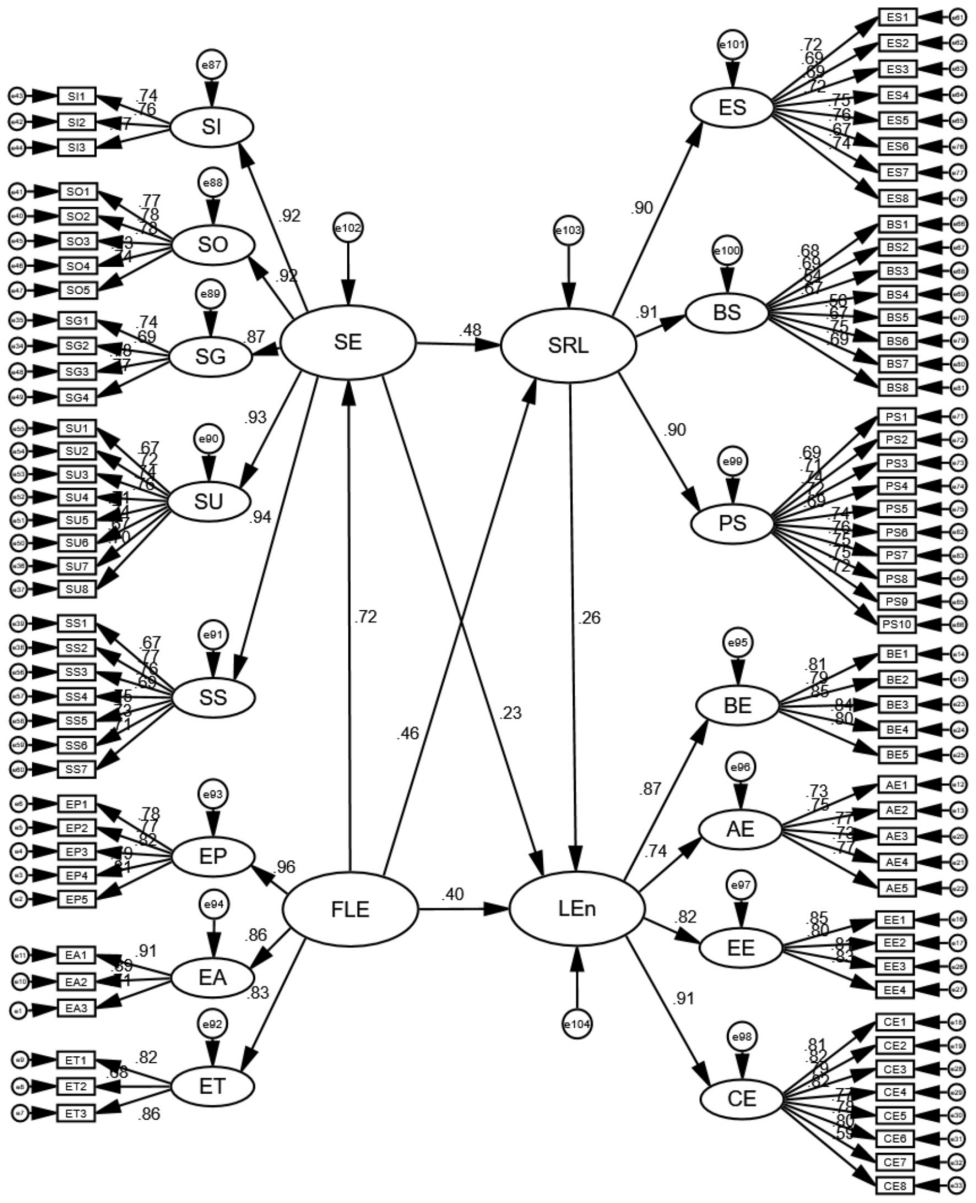
Structural model diagram.

To clarify the structural relationships within the proposed model, this study examined the path coefficients and tested the hypotheses (see Figure 2). The standardized results of the path analysis are summarized in Table 6.

**Table 6:** Structural model path analysis results.

Hypothesis	Relationship	Unstd.	S.E.	*z*-value	*p*-value	Std.	*R* ^2^	Results
H1	FLE → LE	0.393	0.067	5.898	^***^	0.404	0.685	supported
H5	SE → LE	0.287	0.079	3.610	^***^	0.230		supported
H6	SRL → LE	0.281	0.090	3.134	0.002	0.261		supported
H2	FLE → SE	0.559	0.044	12.716	^***^	0.718	0.516	supported
H3	FLE → SRL	0.418	0.048	8.614	^***^	0.464	0.770	supported
H4	SE → SRL	0.559	0.064	8.782	^***^	0.483		supported

LE, learner engagement; SRL, self-regulated learning; SE, self-efficacy; FLE, foreign language enjoyment. ^***^p < 0.001.

All six hypothesized paths were statistically significant, thus providing full support for the proposed model. Specifically, learner engagement was positively influenced by foreign language enjoyment (β = 0.404, *p* < 0.001), self-efficacy (β = 0.230, *p* < 0.001), and self-regulated learning (β = 0.261, *p* = 0.002). In addition, self-efficacy was significantly and positively related to foreign language enjoyment (β = 0.718, *p* < 0.001). Furthermore, self-regulated learning was positively affected by both foreign language enjoyment (β = 0.464, *p* < 0.001) and self-efficacy (β = 0.483, *p* < 0.001). The standardized path coefficients (β) reflect the strength and direction of the relationships among the constructs. For instance, a one-unit increase in FLE corresponds to a 0.404-unit increase in learner engagement, a 0.718-unit increase in self-efficacy, and a 0.464-unit increase in self-regulated learning.

In terms of explanatory power, the model accounted for 68.5% of the variance in learner engagement, 51.6% of the variance in self-efficacy, and 77.0% of the variance in self-regulated learning.

### 4.7 Mediation analysis

To examine the indirect effects of the independent variable on the dependent variable (LE) through the mediators (SE and SRL), a bootstrapping procedure was conducted with 5,000 resamples, generating both percentile and bias-corrected percentile 95% confidence intervals. Following the recommendations of Preacher and Hayes (2008), the significance of the indirect effects was determined by examining the lower and upper bounds of these confidence intervals.

As presented in Table 7, the bootstrap results revealed a positive and significant total indirect effect of SE and SRL (the serial mediation of SE and SRL) in the relationship between FLE and LE, with a point estimate of 0.366 (BC 95% CI [0.249, 0.510]; percentile 95% CI [0.244, 0.505]). Furthermore, the specific indirect effects were also positive and significant: The indirect effect via SE alone was 0.160 (BC 95% CI [0.039, 0.307]; percentile 95% CI [0.036, 0.306]), and the indirect effect via SRL alone was 0.118 (BC 95% CI [0.013, 0.244]; percentile 95% CI [0.005, 0.237]). In addition, the serial mediation path from FLE to LE through SE and SRL in sequence yielded a significant indirect effect of 0.088 (BC 95% CI [0.010, 0.190]; percentile 95% CI [0.004, 0.183]).

**Table 7:** Mediation analysis results.

Relations	Point estimation	Product of coefficients		Bootstrapping						Total%
			BC 95% CI			Percentile 95% CI				
		SE	*Z*	Lower	Upper	*P*	Lower	Upper	*P*	
Total	0.759	0.055	13.800	0.666	0.884	0.000	0.656	0.873	0.001	100.000
Indirect effects										
1.FLE → SE → SRL → LE	0.088	0.045	1.956	0.010	0.190	0.033	0.004	0.183	0.044	11.594
2.FLE → SE → LE	0.160	0.068	2.353	0.039	0.307	0.013	0.036	0.306	0.015	21.080
3.FLE → SRL → LE	0.118	0.058	2.034	0.013	0.244	0.036	0.005	0.237	0.045	15.547
Total indirect	0.366	0.066	5.545	0.249	0.510	0.001	0.244	0.505	0.001	48.221
Direct effects										
FLE → LE	0.393	0.079	4.975	0.256	0.573	0.001	0.241	0.559	0.001	51.779
Contrasts										
1 vs. 2	−0.073	0.104	−0.702	−0.278	0.133	0.451	−0.285	0.130	0.432	–
1 vs. 3	−0.030	0.038	−0.789	−0.130	0.026	0.260	−0.117	0.039	0.392	–
2 vs. 3	0.043	0.116	0.371	−0.173	0.277	0.699	−0.169	0.282	0.680	–

In terms of direct effects, the direct path from FLE to LE remained positive and significant after accounting for the mediators, with a point estimate of 0.393 (BC 95% CI [0.256, 0.573]; percentile 95% CI [0.241, 0.559]), indicating that FLE still exerted a direct influence on LE beyond the mediation pathways. The direct effect accounted for 51.779% of the total effect, further supporting the partial mediation nature of the model.

Regarding the pairwise comparisons of the indirect effects, the contrast analysis revealed the following: The difference between the serial mediation effect (FLE → SE → SRL → LE) and the simple mediation effect via SE alone was negative but not statistically significant (point estimate = −0.073, Z = −0.702, BC 95% CI [−0.278, 0.133], percentile 95% CI [−0.285, 0.130]), indicating that the indirect effect through SE alone did not significantly differ from the indirect effect transmitted through the sequential pathway of SE and SRL. The difference between the serial mediation effect (FLE → SE → SRL → LE) and the simple mediation effect via SRL alone was also not significant (point estimate = −0.030, Z = −0.789, BC 95% CI [−0.130, 0.026], percentile 95% CI [−0.117, 0.039]), as both bias-corrected and percentile confidence intervals contained zero. This suggests that the chain-mediation effect was comparable to the indirect effect via SRL alone. The difference between the two simple mediation effects, SE alone vs. SRL alone, was positive but not statistically significant (point estimate = 0.043, *Z* = 0.371, BC 95% CI [−0.173, 0.277], percentile 95% CI [−0.169, 0.282]), indicating that the indirect effect via SE did not significantly differ in magnitude from that via SRL in linking FLE to LE. Overall, none of the contrasts among the three indirect effects reached statistical significance, suggesting that the three mediation pathways contributed similarly to the overall indirect effect.

## 5 Discussion

### 5.1 Enjoyment and engagement

The results support a partial mediation model. After accounting for self-efficacy and SRL strategies, FLE is still positively associated with learner engagement. This indicates that enjoyment not only operates through cognitive and motivational pathways but also exerts a significant direct influence on engagement.

This result corroborates the well-documented synergy between FLE and learner engagement established in recent literature (Derakhshan and Yin, 2025). This direct pathway aligns with CVT (Pekrun, 2006). In AI-enhanced environments, FLE reflects learners' perceived control and task value, directly sustaining engagement. Specifically, this emotional state optimizes the allocation of cognitive resources, allowing learners to bypass the “affective filter” and engage deeply with the task (Brockbank and Feldon, 2024). According to BBT, positive emotions expand what learners think and do in the moment (Fredrickson, 2004; MacIntyre and Mercer, 2014). According to CVT, enjoyment, as a positive emotion arising from high control and high value, inherently possesses the function of directly promoting learner engagement, without necessarily needing to operate through self-efficacy or SRL strategies to foster engagement. According to BBT, positive emotions can instantly expand individuals' cognitive and behavioral repertoires, enabling learners to more proactively experiment, explore, and persist in AI-assisted writing, thereby directly increasing their level of engagement. Therefore, theoretically, CVT and BBT support the dominant positive influence of enjoyment on learner engagement.

Rather than simply following AI prompts, the “enjoyed” learner is more likely to experiment, play with language, and persist in the writing process—not necessarily because they feel “competent,” but because the positive emotion itself sustains their interest. Consequently, as Pekrun and Linnenbrink-Garcia (2012) suggest, these positive emotions are not merely “side effects” of success, but are fundamental, direct precursors to behavioral and cognitive investment in the writing process.

### 5.2 The mediating role of self-efficacy

The SEM analysis of results reveals a significant positive relationship between FLE and writing self-efficacy within the AI-assisted context. It is because when Chinese university students experience positive emotional activation while utilizing AI writing tools, they concurrently develop stronger subjective beliefs in their academic capabilities. This robustly aligns with a growing consensus in L2 research demonstrating that enjoyment is positively associated with learners' competence beliefs across various domains, including academic writing and listening comprehension (Arfiandhani and Takeuchi, 2025; Kang and Wu, 2022; Solhi and Derakhshan, 2025; Sun et al., 2026; Zhang L. J. et al., 2025). This study confirms the strong correlation between FLE and self-efficacy observed in traditional environments (Oyama, 2022).

This emotional-cognitive transition is best elucidated through the lens of Pekrun's CVT. In an AI-supported environment, FLE serves as an internal feedback loop, and the pleasant emotional state signals to learners that they are effectively managing the writing process, thereby reinforcing their perceived competence and their confidence to tackle future tasks. Consequently, this heightened self-efficacy serves as the primary factor for learner engagement. These constructs are significantly positively related: Learners with stronger self-efficacy beliefs in AI-assisted writing demonstrate higher engagement. This corroborates recent empirical evidence characterizing self-efficacy as a multifaceted factor of cognitive, affective, and behavioral investment (Cong et al., 2024; Derakhshan and Fathi, 2024; Xu et al., 2024; Zare et al., 2025). Notably, within digital and feedback-rich contexts, self-efficacy often emerges as the paramount variable which is positively associated with learner persistence and achievement (Tsao, 2021).

This finding supports the reciprocal relationship proposed by SCT: personal beliefs and digital environments interact dynamically in L2 writing. In a feedback-rich digital environment, self-efficacy serves as the “hub” for the transmission of feedback effects (He and Wen, 2025). Highly self-efficacious learners are more inclined to utilize AI scaffolding to set challenging goals, sustain motivational commitment, and exhibit resilience when facing linguistic setbacks, ultimately facilitating deeper learner engagement. Therefore, this study demonstrates that self-efficacy serves as a crucial mediator between FLE and learner engagement within AI-assisted writing.

Specifically, FLE can be positively related to engagement through a dual mechanism, which includes a direct affective pathway and an indirect cognitive-mediational pathway affected by self-efficacy. This dual mechanism is firmly anchored in educational psychology. Several scholars demonstrated within SRL frameworks that emotions influence academic achievement not only directly but also indirectly by shaping motivational beliefs (Linnenbrink and Pintrich, 2002; Pintrich, 2003). In the context of digital learning, this result corroborates recent empirical evidence. (Wang and Wang 2025a) and (Wang and Zhou 2025b) identified similar mediating roles for self-efficacy between FLE and engagement in AI-assisted and virtual reality-assisted language learning environments, respectively.

Innovatively, this study expands on this foundation by clarifying the psychological mechanisms unique to AI-assisted EFL writing. AI tools offer immediate scaffolding that can transform writing into an enjoyable journey of discovery (Cen and Shakibaei, 2026). Drawing on CVT (Pekrun et al., 2017), the enjoyment is directly positively associated with perceived control over complex writing processes while reinforcing the subjective value they assign to the task, accounting for the direct effect of enjoyment on engagement. This is how the direct pathway is activated. Concurrently, the indirect pathway is activated: When learners experience heightened enjoyment and reduced frustration during AI-assisted tasks, they are more likely to interpret their successful interactions as “mastery experiences” (Bandura, 1997; MacIntyre and Mercer, 2014). These mastery experiences solidify their self-efficacy beliefs regarding their writing capabilities. As this cognitive appraisal improves, it drives deeper learner engagement, manifesting as increased persistence, strategic effort regulation, and sustained cognitive investment (Schunk and DiBenedetto, 2020).

### 5.3 The mediating role of SRL strategies

The result indicates a robust positive relationship between FLE and SRL, demonstrating that when learners experience positive emotion in AI-assisted writing environments, they are significantly more likely to deploy deep, conscious SRL strategies. This corroborates recent findings in digital L2 contexts (Dan and Bai, 2025; Luo and Wang, 2023; Zhao et al., 2023) and can be theoretically elucidated through CVT. As MacIntyre and Mercer (2014) established in the L2 context, positive emotions do not merely make learners feel comfortable. They actively broaden learners' momentary thought-action repertoires. In an AI-assisted environment, this means that enjoyment counteracts the restrictive, tunnel-vision effects of writing anxiety, thereby freeing cognitive capacity to deploy complex, metacognitive SRL strategies (Ahmed et al., 2013; Pekrun et al., 2002).

Specifically, the enhanced self-regulatory capacity translates directly into heightened learner engagement. Aligning with contemporary empirical research (Shi et al., 2025; Wang X. et al., 2025; Zare et al., 2024), the results indicate that learners who consciously apply SRL strategies exhibit proportionally greater behavioral and cognitive investment in the writing task. What's more, in the specific context of AI mediation, learners with strong SRL skills do not passively consume AI outputs. Instead, they interact with these AI tools agentically—setting specific goals, actively seeking targeted feedback, and evaluating alternative linguistic perspectives (Lee D. et al., 2025).

Ultimately, these results converge to establish SRL strategies as a critical mediator between FLE and learner engagement. The “FLE-SRL-engagement” pathway illustrates that positive emotions are positively related to language learning engagement both directly and through an indirect, cognitive pathway. This mediation conceptually parallels the serial pathways identified by Zhao et al. (2023), wherein motivational factors influence L2 writing through the active utilization of strategies. As learners experience the “enjoyment of writing” facilitated by AI, they build the self-regulatory competence necessary to navigate linguistic challenges independently (Xu, 2026). This also reinforces Cleary's (2025) assertion that learner agency is fundamentally anchored in the mastery of self-regulatory processes. In essence, AI-assisted writing fosters a cyclical, empowering dynamic process. Positive affect triggers strategic exploration, which solidifies sustained and deeply agentic engagement.

### 5.4 The distal mediation of self-efficacy and SRL strategies

Overall, this study demonstrates that self-efficacy and SRL act as sequential mediators in the relationship between FLE and learner engagement in AI-assisted writing. The predictive relationship between self-efficacy and SRL is well-documented in educational psychology. DiBenedetto and Bembenutty (2013) empirically supported this motivational-regulatory pathway, while more recent studies have confirmed its relevance across diverse disciplines, including nursing education (Morsy et al., 2025) and computer programming (Li et al., 2025). In technology-enhanced contexts, this relationship is particularly salient. As confirmed by the current findings and aligned with recent L2 research (Chen et al., 2024; Jin et al., 2025; Liu and Zhang, 2025; Teng, 2024; Zhang and Zhang, 2024), self-efficacious learners are better equipped to leverage generative tools strategically. Rather than using AI as a mere cognitive crutch or answer generator, these students employ it as a dialogic partner for metacognitive planning, monitoring, and revision—core points of Zimmerman's (2000) cyclical model of self-regulation.

According to Pekrun's CVT, positive emotions emerge when learners perceive high control and value in a task, which are positively associated with subsequent motivational beliefs. In AI-assisted writing, where intelligent tools offer immediate, non-judgmental scaffolding (Wang X. et al., 2025), learners experience a mastery-oriented atmosphere that mitigates anxiety and amplifies FLE. This affective resource fuels self-efficacy, providing the confidence necessary to enact complex SRL strategies. As students actively apply goal-setting and adaptive help-seeking—such as iterative prompting with AI—they naturally transition into higher states of behavioral, cognitive, and emotional engagement. This sequential chain corroborates recent empirical models, such as Xu's (2026) findings on how “intention to use” sequentially mediates the relationship between AI perceptions and engagement, as well as Liu et al.'s (2025) evidence that AI-mediated environments cultivate the motivational self-concept required for deep learning.

## 6 Limitations

Three limitations should be acknowledged. First, due to its cross-sectional design, this study cannot establish causal relationships among the examined variables. Future research could adopt longitudinal or experimental designs to better infer causality. Second, the sample was exclusively drawn from university students in mainland China, which constrains the cross-cultural applicability of the findings. Subsequent investigations could expand the geographical and cultural coverage, thereby strengthening the generalizability of the proposed model. Third, the sample was drawn primarily from undergraduate students at a single key university in China, which limits the generalizability of the findings. Due to potential differences in cultural attitudes toward artificial intelligence, educational policies, and language learning experiences across diverse populations, the relationships among variables may vary. Therefore, future research should include more diverse populations from different geographical regions and educational stages—such as students from other regions and countries, as well as those at different educational levels, such as primary and secondary school students, graduate students, and vocational college students. Overall, future research should adopt longitudinal or experimental designs to verify causality and dynamic changes over time, include diverse cultural samples to test the stability of the model across different regions and educational contexts, use mixed methods that combine qualitative inquiry and objective behavioral data to improve measurement validity, and conduct in-depth analyses of AI tool types and usage patterns to explore their differential impacts on learner engagement.

## 7 Conclusion

This study investigates the relationships among FLE, self-efficacy, SRL strategies, and learner engagement in AI-assisted EFL writing. A sample of 535 university students from China was recruited. Their questionnaire and data were analyzed using confirmatory factor analysis to establish construct validity, followed by structural equation modeling to test the hypothesized direct and indirect pathways. The results support a well-fitting model in which FLE is positively associated with learner engagement, both directly and indirectly, through self-efficacy and SRL strategies in AI-assisted EFL writing. The non-significant contrasts among the three indirect paths suggest that self-efficacy and self-regulated learning strategies function as functionally equivalent and substitutable mediators in translating foreign language enjoyment into learner engagement. Theoretically, this indicates that positive emotions can channel into engagement through multiple parallel routes. Pedagogically, this finding implies that instructors have flexible intervention options: They may choose to enhance learners' self-efficacy, teach self-regulated learning strategies, combine both approaches, or directly foster learners' enjoyment.

## Data Availability

The raw data supporting the conclusions of this article will be made available by the authors, without undue reservation.
