# Supplementary material for: Fostering engagement in the digital age: the mediating role of self-efficacy and self-regulation between enjoyment and learner engagement in AI-assisted EFL writing
Source: Front Psychol. 2026 Jun 15;17:1848701. doi: 10.3389/fpsyg.2026.1848701 (PMC13311117; doi:10.3389/fpsyg.2026.1848701)
Supplement: Supplementary file 1 [file Supplementary_file_1.docx]

# Appendix A

Table A1 Descriptive statistics.

| **Items** | **M** | **SD** | **Skewness** | **Kurtosis** |
| --- | --- | --- | --- | --- |
| AE1 | 5.090 | 1.363 | -0.451 | -0.217 |
| AE2 | 4.970 | 1.258 | -0.378 | -0.125 |
| AE3 | 5.180 | 1.200 | -0.518 | 0.272 |
| AE4 | 5.180 | 1.204 | -0.485 | 0.017 |
| AE5 | 4.990 | 1.347 | -0.405 | -0.396 |
| BE1 | 5.610 | 1.022 | -0.673 | 0.705 |
| BE2 | 5.410 | 1.079 | -0.407 | -0.018 |
| BE3 | 5.650 | 1.049 | -0.700 | 0.600 |
| BE4 | 5.610 | 1.016 | -0.568 | 0.517 |
| BE5 | 5.550 | 1.046 | -0.526 | 0.389 |
| EE1 | 5.600 | 1.055 | -0.539 | 0.030 |
| EE2 | 5.650 | 1.042 | -0.584 | 0.166 |
| EE3 | 5.610 | 0.946 | -0.585 | 0.858 |
| EE4 | 5.500 | 0.983 | -0.579 | 0.503 |
| CE1 | 5.590 | 0.969 | -0.585 | 0.876 |
| CE2 | 5.610 | 1.025 | -0.716 | 0.842 |
| CE3 | 5.590 | 0.982 | -0.605 | 0.563 |
| CE4 | 5.620 | 1.033 | -0.592 | 0.245 |
| CE5 | 5.550 | 1.015 | -0.474 | 0.403 |
| CE6 | 5.470 | 1.022 | -0.429 | 0.589 |
| CE7 | 5.640 | 1.048 | -0.597 | 0.086 |
| CE8 | 5.640 | 0.960 | -0.152 | -0.763 |
| SI1 | 5.820 | 0.857 | -0.565 | 0.509 |
| SI2 | 5.760 | 0.983 | -0.601 | 0.304 |
| SI3 | 5.680 | 0.944 | -0.360 | -0.282 |
| SG1 | 5.730 | 1.086 | -0.726 | 0.591 |
| SG2 | 5.680 | 0.992 | -0.543 | 0.277 |
| SG3 | 5.790 | 0.964 | -0.640 | 0.454 |
| SG4 | 5.820 | 0.960 | -0.607 | 0.025 |
| SS1 | 5.500 | 0.977 | -0.196 | -0.395 |
| SS2 | 5.820 | 1.006 | -0.735 | 0.251 |
| SS3 | 5.700 | 1.026 | -0.493 | -0.267 |
| SS4 | 5.520 | 0.998 | -0.235 | -0.529 |
| SS5 | 5.830 | 1.001 | -0.670 | 0.112 |
| SS6 | 5.500 | 1.022 | -0.472 | -0.114 |
| SS7 | 5.700 | 0.971 | -0.419 | -0.212 |
| SU1 | 5.590 | 0.923 | -0.395 | 0.231 |
| SU2 | 5.700 | 0.932 | -0.268 | -0.537 |
| SU3 | 5.650 | 0.994 | -0.472 | 0.190 |
| SU4 | 5.610 | 0.952 | -0.284 | -0.334 |
| SU5 | 5.670 | 1.037 | -0.508 | -0.296 |
| SU6 | 5.720 | 0.930 | -0.551 | 0.644 |
| SU7 | 5.530 | 1.077 | -0.485 | -0.102 |
| SU8 | 5.700 | 0.927 | -0.320 | -0.164 |
| SO1 | 5.670 | 0.954 | -0.372 | -0.214 |
| SO2 | 5.710 | 0.924 | -0.337 | -0.318 |
| SO3 | 5.710 | 0.927 | -0.223 | -0.687 |
| SO4 | 5.700 | 0.882 | -0.220 | -0.358 |
| SO5 | 5.730 | 0.938 | -0.387 | -0.166 |
| EP1 | 5.550 | 0.989 | -0.336 | -0.349 |
| EP2 | 5.590 | 1.011 | -0.441 | -0.139 |
| EP3 | 5.700 | 1.001 | -0.505 | 0.019 |
| EP4 | 5.620 | 0.954 | -0.407 | -0.281 |
| EP5 | 5.660 | 1.027 | -0.482 | -0.308 |
| ET1 | 5.840 | 1.042 | -0.842 | 0.607 |
| ET2 | 5.980 | 1.055 | -0.861 | 0.166 |
| ET3 | 5.900 | 1.051 | -0.702 | 0.021 |
| EA1 | 5.490 | 1.106 | -0.400 | -0.432 |
| EA2 | 5.410 | 1.105 | -0.314 | -0.600 |
| EA3 | 5.640 | 1.004 | -0.452 | -0.170 |
| ES1 | 5.680 | 1.027 | -0.482 | -0.373 |
| ES2 | 5.540 | 1.003 | -0.290 | -0.440 |
| ES3 | 5.610 | 0.975 | -0.299 | -0.496 |
| ES4 | 5.560 | 1.081 | -0.540 | -0.135 |
| ES5 | 5.730 | 1.010 | -0.567 | -0.138 |
| ES6 | 5.560 | 1.036 | -0.452 | -0.349 |
| ES7 | 5.820 | 0.962 | -0.514 | -0.217 |
| ES8 | 5.780 | 1.011 | -0.841 | 0.878 |
| PS1 | 5.840 | 1.038 | -0.503 | -0.475 |
| PS2 | 5.770 | 0.910 | -0.301 | -0.504 |
| PS3 | 5.690 | 0.981 | -0.311 | -0.598 |
| PS4 | 5.730 | 0.966 | -0.357 | -0.446 |
| PS5 | 5.790 | 0.977 | -0.578 | -0.014 |
| PS6 | 5.670 | 1.009 | -0.413 | -0.289 |
| PS7 | 5.670 | 0.974 | -0.390 | -0.358 |
| PS8 | 5.700 | 0.978 | -0.364 | -0.495 |
| PS9 | 5.720 | 0.957 | -0.524 | -0.088 |
| PS10 | 5.740 | 1.027 | -0.718 | 0.564 |
| BS1 | 5.710 | 0.961 | -0.300 | -0.523 |
| BS2 | 5.670 | 0.953 | -0.299 | -0.486 |
| BS3 | 5.390 | 1.150 | -0.547 | 0.201 |
| BS4 | 5.640 | 1.085 | -0.626 | 0.126 |
| BS5 | 5.520 | 1.038 | -0.294 | -0.552 |
| BS6 | 5.680 | 0.995 | -0.483 | -0.157 |
| BS7 | 5.780 | 0.957 | -0.452 | -0.313 |
| BS8 | 5.690 | 0.951 | -0.167 | -0.803 |

*Note.* N = 535. AE = agentic engagement; BE = behavioral engagement; EE = emotional engagement; CE = cognitive engagement; SI = self-efficacy for ideation; SG = self-efficacy for grammar and spelling; SS = self-efficacy for self-regulation; SU = self-efficacy for use of English writing; SO = self-efficacy for organization; EP = foreign language enjoyment-Private; EA = foreign language enjoyment-Atmosphere; ET = foreign language enjoyment-Teacher; ES = environmental self-regulated learning strategies; BS = behavioral self-regulated learning strategies; PS = personal self-regulated learning strategies.

**Table A2** Results of the reliability test of the measurement model.

| **Dimension** | **Items** | **Significance estimation** | | | | | **Cronbach’s α** | **McDonald’s ω** | **Guttman’s λ2** |
| --- | --- | --- | --- | --- | --- | --- | --- | --- | --- |
|  |  | **Unstd.** | **S.E.** | **z-value** | **p-value** | **Std.** |  |  |  |
| AE | AE1 | 1.000 |  |  |  | 0.735 | 0.865 | 0.866 | 0.866 |
|  | AE2 | 0.946 | 0.057 | 16.590 | *** | 0.753 |  |  |  |
|  | AE3 | 0.925 | 0.054 | 16.995 | *** | 0.771 |  |  |  |
|  | AE4 | 0.877 | 0.055 | 16.069 | *** | 0.729 |  |  |  |
|  | AE5 | 1.037 | 0.061 | 16.980 | *** | 0.771 |  |  |  |
| BE | BE1 | 1.000 |  |  |  | 0.811 | 0.911 | 0.911 | 0.911 |
|  | BE2 | 1.032 | 0.050 | 20.817 | *** | 0.793 |  |  |  |
|  | BE3 | 1.078 | 0.047 | 23.048 | *** | 0.853 |  |  |  |
|  | BE4 | 1.034 | 0.045 | 22.732 | *** | 0.844 |  |  |  |
|  | BE5 | 1.011 | 0.048 | 21.120 | *** | 0.801 |  |  |  |
| BS | BS1 | 1.000 |  |  |  | 0.683 | 0.856 | 0.856 | 0.856 |
|  | BS2 | 1.002 | 0.069 | 14.500 | *** | 0.690 |  |  |  |
|  | BS3 | 0.954 | 0.082 | 11.629 | *** | 0.544 |  |  |  |
|  | BS4 | 1.105 | 0.078 | 14.086 | *** | 0.668 |  |  |  |
|  | BS5 | 0.884 | 0.074 | 11.929 | *** | 0.559 |  |  |  |
|  | BS6 | 1.010 | 0.072 | 14.051 | *** | 0.666 |  |  |  |
|  | BS7 | 1.100 | 0.070 | 15.721 | *** | 0.755 |  |  |  |
|  | BS8 | 0.997 | 0.069 | 14.472 | *** | 0.688 |  |  |  |
| CE | CE1 | 1.000 |  |  |  | 0.806 | 0.922 | 0.924 | 0.924 |
|  | CE2 | 1.077 | 0.049 | 21.963 | *** | 0.821 |  |  |  |
|  | CE3 | 0.998 | 0.048 | 20.936 | *** | 0.794 |  |  |  |
|  | CE4 | 1.079 | 0.050 | 21.767 | *** | 0.816 |  |  |  |
|  | CE5 | 1.006 | 0.050 | 20.242 | *** | 0.774 |  |  |  |
|  | CE6 | 1.019 | 0.050 | 20.403 | *** | 0.779 |  |  |  |
|  | CE7 | 1.072 | 0.051 | 21.138 | *** | 0.799 |  |  |  |
|  | CE8 | 0.729 | 0.050 | 14.489 | *** | 0.594 |  |  |  |
| EA | EA1 | 1.000 |  |  |  | 0.911 | 0.864 | 0.881 | 0.871 |
|  | EA2 | 0.976 | 0.033 | 29.203 | *** | 0.890 |  |  |  |
|  | EA3 | 0.711 | 0.036 | 19.944 | *** | 0.713 |  |  |  |
| EE | EE1 | 1.000 |  |  |  | 0.853 | 0.892 | 0.893 | 0.893 |
|  | EE2 | 0.921 | 0.042 | 21.839 | *** | 0.796 |  |  |  |
|  | EE3 | 0.855 | 0.038 | 22.587 | *** | 0.814 |  |  |  |
|  | EE4 | 0.902 | 0.039 | 23.113 | *** | 0.826 |  |  |  |
| EP | EP1 | 1.000 |  |  |  | 0.780 | 0.893 | 0.893 | 0.893 |
|  | EP2 | 1.005 | 0.053 | 18.900 | *** | 0.767 |  |  |  |
|  | EP3 | 1.059 | 0.052 | 20.384 | *** | 0.815 |  |  |  |
|  | EP4 | 0.972 | 0.050 | 19.458 | *** | 0.785 |  |  |  |
|  | EP5 | 1.075 | 0.053 | 20.113 | *** | 0.806 |  |  |  |
| ES | ES1 | 1.000 |  |  |  | 0.721 | 0.895 | 0.896 | 0.896 |
|  | ES2 | 0.928 | 0.060 | 15.345 | *** | 0.686 |  |  |  |
|  | ES3 | 0.912 | 0.059 | 15.512 | *** | 0.693 |  |  |  |
|  | ES4 | 1.049 | 0.065 | 16.096 | *** | 0.719 |  |  |  |
|  | ES5 | 1.029 | 0.061 | 16.918 | *** | 0.755 |  |  |  |
|  | ES6 | 1.068 | 0.062 | 17.117 | *** | 0.763 |  |  |  |
|  | ES7 | 0.870 | 0.058 | 14.988 | *** | 0.670 |  |  |  |
|  | ES8 | 1.012 | 0.061 | 16.617 | *** | 0.741 |  |  |  |
| ET | ET1 | 1.000 |  |  |  | 0.819 | 0.824 | 0.829 | 0.826 |
|  | ET2 | 0.836 | 0.052 | 16.183 | *** | 0.676 |  |  |  |
|  | ET3 | 1.056 | 0.050 | 21.114 | *** | 0.858 |  |  |  |
| PS | PS1 | 1.000 |  |  |  | 0.686 | 0.917 | 0.917 | 0.917 |
|  | PS10 | 1.032 | 0.067 | 15.390 | *** | 0.715 |  |  |  |
|  | PS2 | 0.908 | 0.059 | 15.292 | *** | 0.711 |  |  |  |
|  | PS3 | 1.015 | 0.064 | 15.814 | *** | 0.737 |  |  |  |
|  | PS4 | 0.970 | 0.063 | 15.384 | *** | 0.715 |  |  |  |
|  | PS5 | 0.942 | 0.064 | 14.801 | *** | 0.686 |  |  |  |
|  | PS6 | 1.053 | 0.066 | 15.939 | *** | 0.743 |  |  |  |
|  | PS7 | 1.039 | 0.064 | 16.259 | *** | 0.759 |  |  |  |
|  | PS8 | 1.034 | 0.064 | 16.120 | *** | 0.752 |  |  |  |
|  | PS9 | 1.011 | 0.063 | 16.113 | *** | 0.752 |  |  |  |
| SG | SG1 | 1.000 |  |  |  | 0.744 | 0.832 | 0.834 | 0.833 |
|  | SG2 | 0.847 | 0.055 | 15.389 | *** | 0.690 |  |  |  |
|  | SG3 | 0.928 | 0.053 | 17.396 | *** | 0.778 |  |  |  |
|  | SG4 | 0.912 | 0.053 | 17.172 | *** | 0.768 |  |  |  |
| SI | SI1 | 1.000 |  |  |  | 0.735 | 0.801 | 0.803 | 0.802 |
|  | SI2 | 1.193 | 0.071 | 16.918 | *** | 0.764 |  |  |  |
|  | SI3 | 1.161 | 0.068 | 17.145 | *** | 0.775 |  |  |  |
| SO | SO1 | 1.000 |  |  |  | 0.769 | 0.871 | 0.872 | 0.873 |
|  | SO2 | 0.979 | 0.052 | 18.653 | *** | 0.776 |  |  |  |
|  | SO3 | 0.991 | 0.053 | 18.849 | *** | 0.783 |  |  |  |
|  | SO4 | 0.874 | 0.051 | 17.255 | *** | 0.726 |  |  |  |
|  | SO5 | 0.950 | 0.054 | 17.694 | *** | 0.742 |  |  |  |
| SS | SS1 | 1.000 |  |  |  | 0.671 | 0.888 | 0.888 | 0.888 |
|  | SS2 | 1.186 | 0.074 | 16.031 | *** | 0.773 |  |  |  |
|  | SS3 | 1.182 | 0.075 | 15.705 | *** | 0.755 |  |  |  |
|  | SS4 | 1.055 | 0.072 | 14.571 | *** | 0.694 |  |  |  |
|  | SS5 | 1.148 | 0.073 | 15.646 | *** | 0.752 |  |  |  |
|  | SS6 | 1.141 | 0.075 | 15.287 | *** | 0.732 |  |  |  |
|  | SS7 | 1.058 | 0.071 | 14.964 | *** | 0.715 |  |  |  |
| SU | SU1 | 1.000 |  |  |  | 0.666 | 0.891 | 0.891 | 0.891 |
|  | SU2 | 1.083 | 0.073 | 14.863 | *** | 0.715 |  |  |  |
|  | SU3 | 1.192 | 0.078 | 15.272 | *** | 0.738 |  |  |  |
|  | SU4 | 1.175 | 0.075 | 15.657 | *** | 0.760 |  |  |  |
|  | SU5 | 1.204 | 0.081 | 14.840 | *** | 0.714 |  |  |  |
|  | SU6 | 1.114 | 0.073 | 15.249 | *** | 0.737 |  |  |  |
|  | SU7 | 1.174 | 0.084 | 14.041 | *** | 0.670 |  |  |  |
|  | SU8 | 1.054 | 0.072 | 14.566 | *** | 0.699 |  |  |  |
| FLE | EP | 1.000 |  |  |  | 0.964 | 0.933 | 0.933 | 0.934 |
|  | EA | 1.161 | 0.064 | 18.129 | *** | 0.856 |  |  |  |
|  | ET | 0.949 | 0.060 | 15.776 | *** | 0.826 |  |  |  |
| SE | SI | 1.000 |  |  |  | 0.917 | 0.960 | 0.960 | 0.960 |
|  | SO | 1.161 | 0.076 | 15.345 | *** | 0.915 |  |  |  |
|  | SG | 1.220 | 0.085 | 14.348 | *** | 0.873 |  |  |  |
|  | SU | 0.989 | 0.072 | 13.776 | *** | 0.929 |  |  |  |
|  | SS | 1.062 | 0.076 | 13.911 | *** | 0.936 |  |  |  |
| SRL | ES | 1.000 |  |  |  | 0.903 | 0.951 | 0.952 | 0.952 |
|  | BS | 0.895 | 0.066 | 13.580 | *** | 0.912 |  |  |  |
|  | PS | 0.959 | 0.070 | 13.677 | *** | 0.901 |  |  |  |
| LE | BE | 1.000 |  |  |  | 0.871 | 0.952 | 0.951 | 0.953 |
|  | AE | 1.021 | 0.078 | 13.142 | *** | 0.737 |  |  |  |
|  | EE | 1.019 | 0.063 | 16.073 | *** | 0.818 |  |  |  |
|  | CE | 0.987 | 0.058 | 16.880 | *** | 0.913 |  |  |  |
